# Degradation of histone deacetylase 4 via the TLR4/JAK/STAT1 signaling pathway promotes the acetylation of high mobility group box 1 (HMGB1) in lipopolysaccharide‐activated macrophages

**DOI:** 10.1002/2211-5463.12456

**Published:** 2018-06-05

**Authors:** Eun J. Park, Young M. Kim, Hye J. Kim, Ki C. Chang

**Affiliations:** ^1^ Department of Pharmacology Institute of Health Sciences College of Medicine Gyeongsang National University Jinju Korea

**Keywords:** high mobility group box 1, histone deacetylase 4, JAK/STAT, sepsis

## Abstract

High mobility group box 1 (HMGB1) has been proposed as crucial in the pathogenesis of many diseases including sepsis. Acetylation of HMGB1 prevents its entry into the nucleus and leads to its secretion from the cell where it can trigger inflammation. We hypothesized that histone deacetylase 4 (HDAC4) controls the acetylation of HMGB1 in lipopolysaccharide (LPS)‐stimulated RAW264.7 cells via the janus kinase (JAK)/signal transducer and activator of transcription (STAT) pathway. The results showed that LPS treatment promoted the degradation of HDAC4 in a proteasome‐dependent manner, which led to HMGB1 acetylation. In LPS‐activated RAW264.7 cells, treatment with TAK‐242 (a toll like receptor 4 inhibitor) and pyridone 6 (a JAK inhibitor) significantly inhibited HDAC4 degradation and acetylation of HMGB1, and thus prevented secretion of HMGB1. Decreased phosphorylation of STAT1 was also observed. Interestingly, HDAC4 overexpression significantly prevented the acetylation and secretion of HMGB1 in both RAW264.7 cells and isolated murine peritoneal macrophages. We conclude that HDAC4 might be a useful target for the treatment of sepsis.

**Abbreviations**
HDAC4: histone deacetylase 4
HMGB1: high mobility group box 1
HO‐1: heme oxygenase‐1
JAK: janus kinase
LPS: lipopolysaccharide
SIRT1: sirtuin 1
STAT: signal transducer and activator of transcription
TLR: toll like receptor

High mobility group box 1 (HMGB1) has been proposed to be a crucial mediator in the pathogenesis of many diseases, including sepsis, arthritis, cancer, autoimmunity diseases and diabetes 1, 2, 3. A ubiquitous protein, it plays a role in DNA replication, recombination, transcription and repair in the nucleus. Targeting HMGB1 may offer a useful novel approach for the treatment of sepsis 4, 5. After translocation to the outside, HMGB1 operates as a warning signal that induces inflammation. Extracellular HMGB1 recognizes multiple receptors, including toll like receptor 2/4 (TLR2/4) and receptor for advanced glycation end products, on immune cells propagating cascades that mediate inflammation 6, 7, 8. Indeed, TLR4 activation promotes the release of HMGB1 from macrophages 9. HMGB1 can be released to the cell exterior via active and passive routes occurring in both somatic and immune cells. Receptor‐mediated HMGB1 release from stimulated cells initially requires hyperacetylation of multiple lysines present in the two HMGB1 nuclear localization sites leading the nuclear HMGB1 to accumulate in the cytoplasm. HMGB1 constantly shuttles between the cytoplasm and the nucleus in a resting cell, but acetylation of lysines in the nuclear localization sites prevents nuclear re‐entry 10, 11. Nuclear acetylation can be accomplished via increased histone acetylase activity as well as decreased histone‐deacetylase (HDAC) activity, partly regulated via janus kinase (JAK)/signal transducer and activator of transcription (STAT) 1 signaling under interferon‐β control. We previously demonstrated that carbon monoxide (CO) inhibited interferon‐β/JAK2/STAT1 activation, which blocked HMGB1 release in lipopolysaccharide (LPS)‐activated RAW264.7 cells 12. These findings indicated that the JAK/STAT1 signaling pathway is important for the post‐translational modification (acetylation of lysine) of HMGB1 in LPS‐induced RAW264.7 cells. Moreover, we found that up‐regulation of sirtuin 1 (SIRT1) by ethyl pyruvate inhibited the LPS‐activated acetylation of HMGB1 by decreasing STAT1 phosphorylation in RAW264.7 cells, confirming that JAK/STAT signaling plays a critical role in the active secretion of HMGB1 by TLR4 activated macrophages 13. Interestingly, the acetylation of HMGB1 is modulated by HDACs in various diseases, such as liver and cerebral ischemia–reperfusion injury and neuroimmune inflammation 14, 15, 16. For example, hyperacetylation of HMGB1 by inhibition of HDAC1 and ‐4 promoted its migration to the extracellular space in different cells 14, 16. However, recently activation of HDAC was found to also inhibit HMGB1 release in human intestinal epithelial HT‐29 cells 17. Therefore, the role (activation or inhibition) of HDACs in HMGB1 release in pro‐inflammatory mediator‐activated cells is controversial. Importantly, it is not yet clear if activation of HDAC4 plays a role in inhibiting the acetylation of HMGB1. Therefore, we hypothesized that activation of HDAC4 might play a crucial role in inhibiting the acetylation of HMGB1 via the TLR4/JAK/STAT1 signaling pathway in LPS‐induced RAW264.7 cells and murine peritoneal macrophages. The present study aimed to investigate the association between HDAC4 and the TLR4/JAK/STAT1 signaling pathway in HMGB1 acetylation in LPS‐activated macrophages.

## Materials and methods

### Materials

HyClone high glucose Dulbecco's modified Eagle's medium (DMEM), FBS, and other tissue culture reagents were purchased from Thermo Fisher Scientific (Waltham, MA, USA). LPS and MG‐132 were purchased from Sigma‐Aldrich (St Louis, MO, USA). TAK‐242 was purchased from Calbiochem (La Jolla, CA, USA). Pyridone 6 was purchased from Cayman Chemical Company (Ann Arbor, MI, USA). Primary antibodies for acetyl‐Lys and HDAC4 and secondary antibodies used for western blot analyses were purchased from Santa Cruz Biotechnology (Dallas, TX, USA). Primary antibodies for phospho‐STAT1 and STAT1 were purchased from Cell Signaling Technology (Danvers, MA, USA). Antibodies for β‐actin and HMGB1 were from Sigma‐Aldrich and Abcam (Cambridge, MA, USA), respectively.

### Cell culture

RAW264.7 macrophages were obtained from the ATCC (Manassas, VA, USA). The cells were maintained at 5 × 10^5^ cells·mL^−1^ in DMEM supplemented with 10% FBS, penicillin (100 units·mL^−1^), streptomycin (100 mg·mL^−1^), l‐glutamine (4.5 mg·mL^−1^) and glucose (4.5 mg·mL^−1^), and incubated at 37 °C in a 5% CO_2_ and 95% air humidified atmosphere.

### Collection of peritoneal macrophages

BALB/c mice (7–8 weeks) were injected with 3% thioglycollate (3 mL, i.p.) for 3 days before peritoneal macrophages were harvested. Murine peritoneal macrophages were harvested with 6 mL of cold Hanks’ balanced salt solution (Wako Pure Chemical Industry, Osaka, Japan). The cells were washed twice and suspended in culture medium [DMEM supplemented with penicillin (100 units·mL^−1^), streptomycin (100 mg·mL^−1^), l‐glutamine (4.5 mg·mL^−1^) and glucose (4.5 mg·mL^−1^)] at a concentration of 10^6^ cells·mL^−1^. Cells were incubated for 2 h at 37 °C in a 5% CO_2_ atmosphere and were washed with PBS to remove non‐adherent cells. Then the adherent cells were cultured with fresh media [DMEM supplemented with penicillin (100 units·mL^−1^), streptomycin (100 mg·mL^−1^), L‐glutamine (4.5mg·mL^−1^) and glucose (4.5 mg·mL^−1^)].

### Transfection

Transient transfections with HDAC4 expression vector, RAW264.7 cells and peritoneal macrophages were performed using the transfection reagent Lipofectamine 2000 (Thermo Fisher Scientific). Cells were seeded in 60 mm plates at 5 × 10^5^ cells/plate prior to transfection and grown to approximately 70% confluence. The cells were transfected with pcDNA 3.1 (1 μg) or the HDAC4 vector (1 μg) for 12 h and then stimulated with LPS (1 μg·mL^−1^). After incubation, cell media and lysates were collected and analyzed by western blotting.

### Ethics statement

Mice were maintained in accordance with the *Guide for the Care and Use of Laboratory Animals* (NIH publication 85‐23, revised 1996) and were treated ethically. The protocol was approved in advance by the Animal Research Committee of the Gyeongsang National University.

### Western blot analysis

After cells were treated with reagents, cells and media were collected for western blot analysis. Total protein from the cells was obtained using RIPA buffer containing 0.5% SDS, 1% Nonidet P‐40, 1% sodium deoxycholate, 150 mm NaCl, 50 mm Tris/Cl (pH 7.5) and protease inhibitor. Total protein concentrations were determined using the Bradford assay. The absorbance of the mixture at 595 nm was determined using an ELISA plate reader. An equal amount of protein from each sample was mixed with sample buffer and boiled. The samples were electrophoresed on 7–12% polyacrylamide gels, followed by transfer onto poly(vinylidene difluoride) western blotting membranes (Roche, Basel, Switzerland). Each membrane was blocked with 5% skim milk and sequentially incubated with a primary antibody and a horseradish peroxidase‐conjugated secondary antibody. Protein bands were visualized by ECL detection (Bio‐Rad Inc., Hercules, CA, USA).

### Immunoprecipitation

For immunoprecipitation, cells were lysed using immunoprecipitation lysis buffer (Thermo Fisher Scientific). After protein extraction, the proteins were precleared with Protein G Sepharose 4 Fast Flow (GE Healthcare Life Sciences, Little Chalfont, UK) for 1 h at 4 °C; the precleared lysate was incubated with anti‐HMGB1 antibody overnight at 4 °C. The precleared lysates were incubated with Protein G Agarose for 4 h. The beads were washed with PBS and western blot analyses were performed.

### Statistical evaluation

Statistics were determined using sigmaplot software (Systat Software, Inc., San Jose, CA, USA). Comparisons of each group were conducted by Student's *t*‐test. Differences between data sets were assessed by one‐way analysis of variance followed by the Newman–Keuls test. Data are expressed as the mean ± SEM. *P *<* *0.05 was accepted as statistically significant.

## Results and Discussion

### LPS induces the acetylation and secretion of HMGB1 though TLR4/JAK/STAT1 signaling

**Figure 1:**
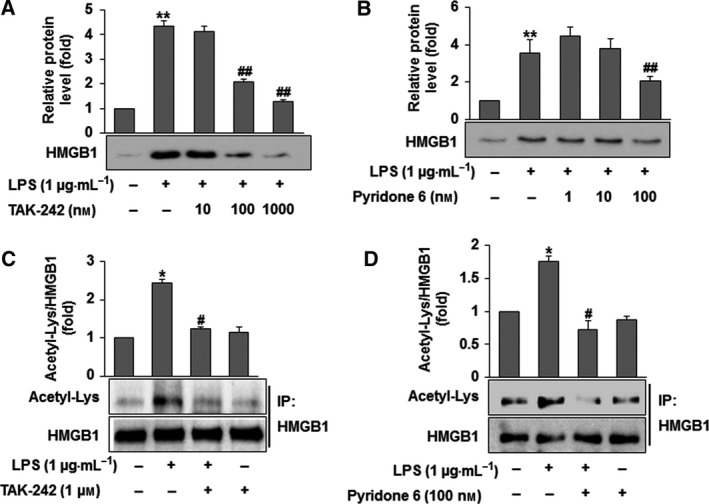
Acetylation and secretion of HMGB1 through TLR4/JAK/STAT1 signaling in LPS‐activated RAW264.7 cells. (A,B) Cells were treated with TAK‐242 (10, 100 or 1000 nm) (A) or pyridone 6 (1, 10 or 100 nm) (B) for 30 min prior to LPS (1 μg·mL ^−1^) treatment. After incubation for 12 h, medium was collected for the detection of HMGB1. Equal volumes of medium were subjected to western blot analysis using an anti‐HMGB1 antibody. (C,D) Cells were treated with TAK‐242 (1 μm) (C) or pyridone 6 (100 nm) (D) for 30 min prior to LPS (1 μg·mL ^−1^) treatment. After incubation for 4 h, cell lysates were subjected to immunoprecipitation using an anti‐HMGB1 antibody and then to western blot analysis using an anti‐acetyl‐Lys antibody. The results are expressed as the means ± SEM of three independent experiments. * *P *<* *0.05 or ** *P *<* *0.01 compared with the controls. ^**#**^ *P *<* *0.05 or ^**##**^ *P *<* *0.01 compared with LPS treatment.

**Figure 2:**
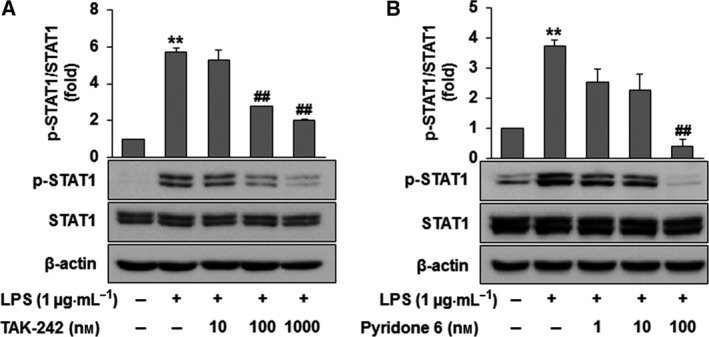
Phosphorylation of STAT1 through TLR4 signaling in LPS‐activated RAW264.7 cells. (A,B) Cells were treated with TAK‐242 (10, 100 or 1000 nm) (A) or pyridone 6 (1, 10 or 100 nm) (B) for 30 min prior to LPS (1 μg·mL ^−1^) treatment. After incubation for 4 h, cell lysates were subjected to western blot analysis using anti‐p‐STAT1 and STAT1 antibodies. The results are expressed as the means ± SEM of three independent experiments. ** *P *<* *0.01 compared with the controls. ^**##**^ *P *<* *0.01 compared with LPS treatment.

It has been recognized that post‐translationally modified HMGB1 is exported from the nucleus to the cytoplasm by chromosomal maintenance 1 (CRM1) and then secreted by lysosome‐mediated exocytosis in LPS‐activated RAW264.7 cells. For the active secretion of HMGB1 in LPS‐activated RAW264.7 cells, JAK/STAT signaling has been shown to be an essential step 12, 13, 18. To confirm the involvement of TLR4/JAK/STAT1 signaling in the LPS‐activated acetylation of HMGB1 in RAW264.7 cells, western blot analyses were performed using specific pharmacological inhibitors. Cells were treated with different concentrations of a TLR4 inhibitor (TAK‐242; 10, 100, 1000 μm) or a JAK inhibitor (pyridine 6; 1, 10, 100 μm) 30 min prior to LPS and incubated for 12 h. We found that HMGB1 release was significantly and concentration‐dependently reduced (Fig. 1A,B). As shown in Fig. 1C,D, inhibitors of TLR4 and JAK also significantly inhibited HMGB1 acetylation in LPS‐stimulated RAW264.7 cells. As shown in Fig. 2A,B, the phosphorylation of STAT1 by LPS was also ablated by treatment with the TLR4 inhibitor and the JAK inhibitor in a concentration‐dependent manner. These findings confirm that the acetylation of HMGB1 in RAW264.7 cells by LPS via TLR4/JAK/STAT signaling is one of the post‐translational modification processes of HMGB1 required for active secretion 12, 18.

### LPS treatment induces HDAC4 degradation through TLR4/JAK/STAT1 signaling

**Figure 3:**
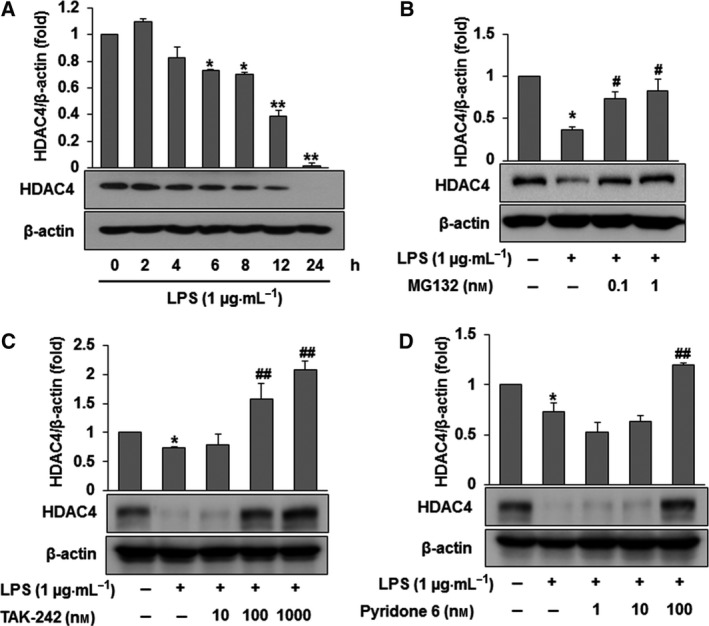
LPS treatment induces HDAC4 loss via proteasome‐dependent degradation in RAW264.7 cells. (A) Cells were treated with LPS (1 μg·mL ^−1^) and then incubated for 0, 2, 4, 6, 8, 12 or 24 h. After incubation for the indicated times, the cell lysates were subjected to western blot analysis using an anti‐HDAC4 antibody. (B) Cells were treated with MG132 (0.1 or 1 μm) for 30 min and then with LPS (1 μg·mL ^−1^). After incubation for 12 h, cell lysates were subjected to western blot analysis using an anti‐HDAC4 antibody. (C) Cells were treated with TAK‐242 (10, 100 or 1000 nm) for 30 min and then treated with LPS (1 μg·mL ^−1^) for 12 h. (D) Cells were treated with pyridone 6 (1, 10 or 100 nm) for 30 min prior to LPS (1 μg·mL ^−1^) treatment. After incubation for 12 h, cell lysates were subjected to western blot analysis using an anti‐HDAC4 antibody. The results are expressed as the means ± SEM of three independent experiments. * *P *<* *0.05 or ** *P *<* *0.01 compared with the controls. ^**#**^ *P *<* *0.05 or ^**##**^ *P *<* *0.01 compared with LPS treatment.

As shown here, TLR4 signaling is associated with a specific downstream pathway that includes the JAK/STAT pathway 9. LPS commonly activates TLR4 and JAK signals and sequentially phosphorylates STAT1, resulting in the release of HMGB1. Thus, JAK/STAT1 signaling is very important for the release of HMGB1 18. Although the activation of JAK/STAT1 by LPS induced HMGB1 cytoplasmic accumulation for subsequent release in LPS‐activated macrophages, it is unclear whether HDAC4 is involved in the acetylation of HMGB1 via JAK/STAT pathways. To determine if there was a link between HDAC4 and the acetylation of HMGB1 in LPS‐activated RAW264.7 cells, the fate of HDAC4 was investigated after LPS treatment. As shown Fig. 3A, the protein expression of HDAC4 decreased in a time‐dependent manner after LPS treatment, which was reversed by MG132, a proteasome inhibitor (Fig. 3B), suggesting that LPS treatment enhanced HDAC4 loss by proteasome‐dependent degradation. HDAC4 loss is presumably linked to the acetylation and active secretion of HMGB1 in the presence of LPS in RAW 264.7 cells. Since HDAC4 is shuttled from the nucleus to the cytoplasm, promoting the acetylation of HMGB1 in liver injury 16, it can be speculated that HDAC4 might maintain HMGB1 in a resting state in the nucleus. However, when the degradation of HDAC4 occurs via LPS. HDAC4 no longer sequesters HMGB1 in the nucleus, which renders exposed HMGB1 unprotected from post‐translational modification. In fact, HDAC4 physically binds to HMGB1 in the nucleus in the resting state but dissociates in the presence of LPS in RAW264.7 cells. Therefore, post‐translational modification of HMGB1 cannot occur as long as HDAC4 binds to HMGB1. Thus, it seems likely that acetylation of HMGB1 in LPS‐activated RAW264.7 cells depends not only on HDAC activity, but also on other factors such as binding of HDAC4 with HMBG1 (considered in a unpublished data). In a recent study, we demonstrated that ethyl pyruvate enhanced the protein expression of SIRT1 to inhibit the phosphorylation of STAT1, causing the suppression of HMGB1 acetylation and thus inhibiting the active secretion of HMGB1 in LPS‐stimulated RAW264.7 cells 13. Because SIRT1, also known as NAD‐dependent deacetylase, epigenetically regulates the acetylation of HMGB1, HDACs may modulate the acetylation of HMGB1. Interestingly, Hwang *et al*. 19 reported that SIRT1 interact with HMGB1 in the nucleus, strongly supporting our present results. In addition, to elucidate whether the degradation of HDAC4 by LPS is associated with TLR4/JAK/STAT1 signaling, TLR4 inhibitor and the JAK inhibitor were exploited. As shown in Fig. 3C,D, HDAC4 degradation by LPS was antagonized in a concentration‐dependent manner by treatment with the TLR4 inhibitor and the JAK inhibitor. These findings indicated that activation of TLR4/JAK/STAT1 signaling by LPS promotes HDAC4 degradation. Here, we showed the association of HDAC4 and the TLR4/JAK/STAT1 signaling pathway in the acetylation of HMGB1 in LPS‐activated RAW264.7 cells. Although the TLR4/JAK/STAT1 signaling pathway has been shown to release HMGB1, this is the first report that the association between this signaling pathway and HDAC4 degradation is linked with the acetylation of HMGB1 in LPS‐activated RAW264.7 cells. It should be noted that ethyl pyruvate also induces the expression of heme oxygenase‐1 (HO‐1) in RAW264.7 cells 20. Previously, we reported that HO‐1 reduced the release of HMGB1 by inhibiting the JAK/STAT1 signaling pathway. Therefore, HO‐1 acts as a negative regulator of HMGB1 release in LPS‐activated RAW264.7 cells. As shown here, crosstalk between the JAK/STAT signaling pathway and HDAC4 degradation may be a critical factor in post‐translational modification of HMGB1. In this context, whether HO‐1 or SIRT1 affects inhibition of HDAC4 degradation that leads to acetylation of HMGB1 in LPS‐activated RAW264.7 cells needs further study.

### HDAC4 reduces HMGB1 acetylation and secretion

**Figure 4:**
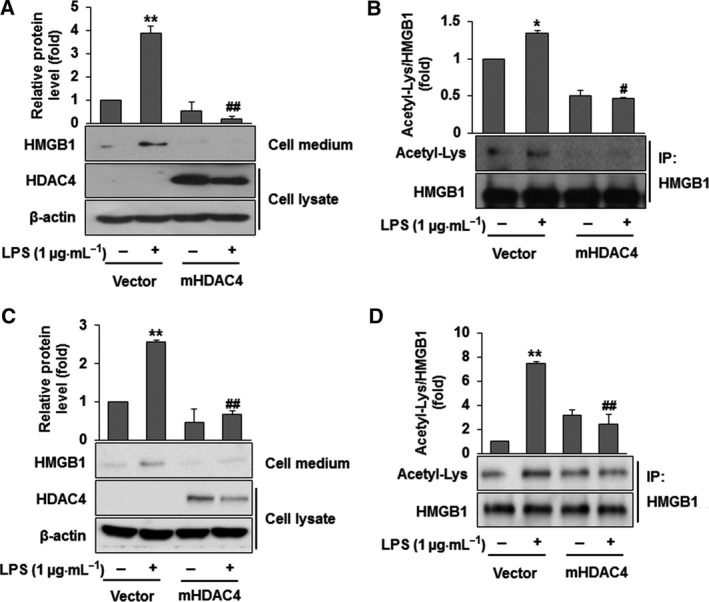
HDAC4 reduces the acetylation and secretion of HMGB1 in LPS‐stimulated RAW264.7 cells and peritoneal macrophages. (A,C) RAW264.7 cells (A) or peritoneal macrophages (C) were transfected with pcDNA 3.1 (1 μg) or the HDAC4 vector (1 μg) for 12 h and then treated with LPS (1 μg·mL ^−1^) for 12 h. After incubation, cell medium and lysates were collected for measuring HMGB1 and HDAC4. Equal volumes of medium were subjected to western blot analysis using an anti‐HMGB1 antibody. Cell lysates were subjected to western blot analysis using an anti‐HDAC4 antibody. (B,D) RAW264.7 cells (B) or peritoneal macrophages (D) were transfected with pcDNA 3.1 (1 μg) or the HDAC4 vector (1 μg) for 12 h prior to LPS (1 μg·mL ^−1^) treatment. After incubation for 4 h, cell lysates were subjected to immunoprecipitation using an anti‐HMGB1 antibody and then to western blot analysis using an anti‐acetyl‐Lys antibody. The results are expressed as the means ± SEM of three independent experiments. * *P *<* *0.05 or ** *P *<* *0.01 compared with the controls. ^**#**^ *P *<* *0.05 or ^**##**^ *P *<* *0.01 compared with LPS treatment.

Finally, the effects of the acetylation and secretion of HMGB1 were investigated in cells that overexpressed HDAC4 protein. We found that overexpression of HDAC4 significantly reduced HMGB1 acetylation and secretion compared with control vector‐transfected cells in the presence of LPS (Fig. 4A,B). Interestingly, LPS treatment reduced HDAC4 expression in HDAC4‐overexpressing RAW264.7 cells (Fig. 4A). To make sure this result is also applicable *ex vivo*, we isolated murine peritoneal microphages and obtained similar results (Fig. 4C,D), indicating that LPS promoted the degradation of HDAC4 in both the established macrophage cell line (RAW264.7 cells) and murine peritoneal macrophages. These findings indicate that HDAC4 no longer maintains HMGB1 in the nucleus in the presence of LPS. Therefore, HMGB1 is liable to be post‐translationally modified owing to an increased chance of exposure resulting from decreased HDAC4 in the nucleus when LPS is present. Indeed, HDAC4 seems to play an important role in controlling shuttling of HMGB1 by an acetylation process in macrophages.

Histone acetylase and HDAC enzymes can mutually regulate the acetylation status of HMGB1 because increased histone acetylase or the inhibition of HDAC results in the acetylation of HMGB1. It has been reported that the suppression of nuclear HDAC activity increases the acetylation of HMGB1 after oxidative stress in hepatocytes and liver ischemia–reperfusion injury 16. Although we did not investigate HDAC4 activity in the present study, it is unlikely that HDAC4 activity is critical for the acetylation of HMGB1 in LPS‐activated RAW264.7 cells. Because we found that overexpression of HDAC4 prevented the acetylation of HMGB1 in LPS‐activated RAW264.7 cells, HDAC4 may interact with HMGB1 independently of its activity in the nucleus as supported by a recent report by Hwang *et al*. 19. Not only HDAC4 but also HDAC1 and HDAC5 have been shown to be important in HMGB1 release from hepatocytes and in brain injury 14, 15, 16. Therefore, it seems likely that distinct types of HDACs play an important role in HMGB1 acetylation in different organs and different types of injury. Indeed, we found that HDAC4 degradation significantly increased the acetylation of HMGB1 in LPS‐activated RAW264.7 cells and that this was strongly associated with the JAK/STAT signaling pathway. To the best of our knowledge, this is the first study to demonstrate that acetylation of HMGB1 is stongly associated with HDAC4 degradation via the TLR4/JAK/STAT1 signaling pathway by LPS in RAW264.7 cells. In other words, the post‐translational modification of HMGB1 depends on degradation of HDAC4 in RAW264.7 cells via JAK/STAT pathways. In summary, we demonstrated that the degradation of HDAC4 by LPS promotes the acetylation and secretion of HMGB1 via the TLR4/JAK/STAT1 signaling pathway in RAW264.7 cells. This result was based on the following findings: (a) acetylation of HMGB1 was dependent on the TLR4/JAK/STAT1 signaling pathway; (b) LPS treatment promoted HDAC4 loss via proteasome‐dependent degradation through the TLR4/JAK/STAT1 signaling pathway; and (c) HDAC4 overexpression inhibited HMGB1 acetylation and release not only in LPS‐activated RAW264.7 cells but also in isolated murine peritoneal macrophages. Therefore, HDAC4 might be a useful target, at least in part, for the treatment of sepsis.

## Author contributions

EJP: performing of experiments, analysis of data. YMK: analysis of data, data collection. HJK: critical reading and writing of the manuscript. KCC: design and writing of the manuscript.
